# Nanoscale Imaging Reveals a Tetraspanin-CD9 Coordinated Elevation of Endothelial ICAM-1 Clusters

**DOI:** 10.1371/journal.pone.0146598

**Published:** 2016-01-05

**Authors:** Jonas Franz, Benjamin F. Brinkmann, Michael König, Jana Hüve, Christian Stock, Klaus Ebnet, Christoph Riethmüller

**Affiliations:** 1 Medical faculty, University of Münster, Münster, Germany; 2 Institute-associated Research Group "Cell adhesion and cell polarity", Institute of Medical Biochemistry, ZMBE, 48419 Münster, Germany; 3 Fluorescence Microscopy Facility Münster, Institute of Medical Physics and Biophysics, Center for Nanotechnology, University of Münster, Münster, Germany; 4 Centre for Internal Medicine, Hannover Medical School, Hannover, Germany; 5 Serend-ip GmbH, Centre for Nanotechnology, Münster, Germany; University of Illinois at Chicago, UNITED STATES

## Abstract

Endothelial barriers have a central role in inflammation as they allow or deny the passage of leukocytes from the vasculature into the tissue. To bind leukocytes, endothelial cells form adhesive clusters containing tetraspanins and ICAM-1, so-called endothelial adhesive platforms (EAPs). Upon leukocyte binding, EAPs evolve into docking structures that emanate from the endothelial surface while engulfing the leukocyte. Here, we show that TNF-α is sufficient to induce apical protrusions in the absence of leukocytes. Using advanced quantitation of atomic force microscopy (AFM) recordings, we found these structures to protrude by 160 ± 80 nm above endothelial surface level. Confocal immunofluorescence microscopy proved them positive for ICAM-1, JAM-A, tetraspanin CD9 and f-actin. Microvilli formation was inhibited in the absence of CD9. Our findings indicate that stimulation with TNF-α induces nanoscale changes in endothelial surface architecture and that—via a tetraspanin CD9 depending mechanism—the EAPs rise above the surface to facilitate leukocyte capture.

## Introduction

The transmigration of leukocytes through an endothelial barrier is a hallmark of inflammation. The process of invasion into the inflamed tissue is a cascade of phases termed rolling, tethering, (firm) adhesion and diapedesis [1, 2]. The first three steps are well characterized by involvement of membrane proteins of the selectin-, IgSF CAM- (cell-adhesion molecule) and integrin- families [3, 4], whereas the mechanism triggering the conversion from a firm adhesion into an actual diapedesis step is less clear.

Closer inspection of the firm adhesion site reveals a close intertwining of membrane structures of both cell partners [5]. Leukocytes palpate the ground for a suitable entry site using so called invadopodia [6, 7], while endothelial cells form a transmigratory cup (TMCup) [8] and propel a corona of filopodial protrusions around the attached leukocyte, which are called docking structure [9, 10]. Docking structures are intensely correlated to the clustering of intercellular adhesion molecule-1 (ICAM-1). The ICAM-1 clustering is mediated by tetraspanins CD9 and CD81 [11, 12]. Among the regulators of the above-mentioned cytoskeletal rearrangements are filamin [13] and the small GTPases RhoA and RhoG [10, 14]. Also guanine nucleotide exchange factors (GEF) like Trio, act downstream of the firm adhesion phase and ICAM-1 clustering, by recruiting β-actin and myosin II—hinting at a contribution of cytomechanics [15, 16].

Following RhoA and RhoG activation, the endothelial cytoskeleton is rearranged, as this is necessary for both paracellular (opening of the junctions) and even more for transcellular pathways (directly through the endothelial cell). For the long-known transcellular way, the endothelial cytoskeleton has to soften locally (depolymerize) to enable the generation of a transmigratory channel [17–21]. A local softening of the endothelial cell directly underneath the leukocyte was shown recently [22, 23].

In a previous in-vitro study of human umbilical vein endothelial cells (HUVECs), we found endothelial activation by TNF-α to be not only a prerequisite, but rather a sufficient condition for a maximum effect on transmigration rate [24]. This favors the endothelium to ultimately decide about allowing or denying the passage of leukocytes. Not only transmigration rate, but also the lateral movement of leukocytes was stimulated by TNF-α. Conclusively, attached leukocytes might be guided laterally to preferred exit sites, where adhesion and diapedesis are facilitated.

After TNF-α activation moesin and ICAM-1 colocalize in the apical membrane at focal conglomerations [25]. Since the ezrin-radizin-moesin complex is crucial in the formation of microvilli in the endothelium, the authors concluded that these conglomerations might be ICAM-1 enriched microvilli [26]. As early as 1998, Heiska et. al. proposed an elevation of ICAM-1 above the cell surface to enhance the binding probability, but did not explicitly determine a protrusion [27].

Here we searched for microvilli on TNF-α-activated human endothelia (HUVEC), that might indicate predilection sites for leukocyte passage. Atomic force microscopy (AFM) was employed to deliver nanometer resolution even under physiological buffer conditions. We found membrane protrusions on endothelial cells being up regulated after activation, prior to any leukocyte contact. These f-actin positive structures synergize with the formation of adhesive ICAM-1 clusters highly dependent on tetraspanin CD9.

## Materials and Methods

### Ethics statement

The University of Muenster (ethics committee: “Ethik-Kommission der Ärztekammer Westfalen-Lippe und der Medizinischen Fakultät der Westfälischen Wilhelms-Universität Münster”) approved the procedures for obtaining umbilical cords from patients and the subsequent isolation of human umbilical endothelial cells (HUVEC) from these samples. The participants provided informed consent in writing before donating the umbilical cords. No genetic information is obtained from the samples and they are collected anonymously. The authors had no contact with the patients and had no access to any identifying information.

### Cell isolation and culture

Veins of fresh human umbilical cords were filled with Dispase-solution (Roche, Mannheim) in Endothelial Growth Medium 1 (Promocell, C-22010) and incubated at 37°C for 15 min. Cells were centrifuged, cultivated up to passage 4 and seeded onto fibronectin-coated cover-slips in *Endothelial Growth Medium 1*.

### Cell treatment

HUVEC were grown to confluence before further treatment. For endothelial activation TNF-α (human recombinant, ImmunoTools 11343015) [10 ng/ml] was added 24 hours before fixation and microscopic analysis.

### siRNA knock-down

HUVECs (2 × 10^6^) were transfected with 200 pmol of siRNAs by nucleofection (Nucleofector Kit; Lonza, Cologne, Germany). After 48–72 h, cells were harvested and analyzed. For depletion of CD9, the following sequence was used: 5-GACGUACUCGAAACCUUCTT-3 [28]. Control cells were transfected with non-targeting siRNA (ON-TARGETplus Non-targeting siRNA #1; Thermo Fisher, Schwerte, Germany).

### Western blotting

CD9 and α-tubulin expression was analyzed using the Odyssey imaging system (LI-COR Biosciences, Bad Homburg, Germany) with the following antibodies: IRDye 800CW donkey anti-Mouse (LI-COR Biosciences, Bad Homburg, Germany), mouse mAb anti–α-tubulin (Sigma, München, Germany) and mouse monoclonal antibody (mAb) anti-CD9 (Millipore, Billerica, MA).

### Atomic force microscopy

For recording the nanoscale surface topography, cell samples were fixed with glutardialdehyde (final conc. 1%, 15 min) added directly into culture dish with growth medium, followed by washing with Phosphate Buffered Saline with Mg and Ca (PBS++) (Dulbecco) and storage at 4°C. They were imaged in PBS++ using an Asylum research MFP 3D: 820 (Mannheim, Germany) in contact mode at forces below 10 nN. Gold coated soft cantilevers with a spring constant of k = 0,08 N/m were used (PNP-TR 50, Nanoworld, Wetzlar, Germany). Maximum tip speed was 70 μm/s. Lateral resolution was 39 nm per pixel.

The analysis procedure for stiffness measurements gently indents the sample in an array of 64*64 spots. The Young‘s moduli are redisplayed as a greyscale map. Black represents softest sample areas—in contrast to the topographical maps, where dark colors code for low-lying regions.

### AFM-Topography analysis

Of each sample, 10 arbitrarily chosen areas of 400–2500 μm^2^ were recorded. Surface object counting (nAnostic^™^ method) was performed using proprietary algorithms for AFM-images (Serend-ip GmbH, Munster, Germany). Each nano-object is characterized by individual size (local deviational volume, LDV) and shape. Basically, the experimentators train an artificial neuronal network with examples of desired structures (machine learning) and then, this pattern is applied to all AFM-recordings. Shown are the object number (n) and their sum LDV per image. The color design was created with the freeware Gwyddion 2.26 (http://gwyddion.net/).

### Immunostaining

The cells were fixed in 4% paraformaldehyde in PBS++ for 15 minutes and washed three times with PBS++. After blocking (0.2% Triton X-100, 0.05% Tween 20, 1% FCS, 0.02% BSA) for 1 hour at RT, the cells were incubated with primary antibodies for either 1 h at RT or overnight at 4°C followed by three washings with PBS++. Afterwards they were incubated with the fluorochrome-conjugated antibody for 2 hours at RT. Optionally, f-actin was stained with Alexa Fluor^®^ 488 phalloidin (Invitrogen, A12379) 1:40 or DNA was stained with DAPI (Sigma, Deisenhofen, Germany). Samples were mounted either in PBS++ or in mounting medium (Dako, Eching, Germany).

#### 4Pi microscopy sample preparation

Cells were covered with 15 μl of MOWIOL 4–88 (CalBIOCHEM) with 4% propyl gallate (Sigma) as anti fade reagent and sealed by a second cover slip with immobilized red fluorescent beads of subresolution size (TransFluoSpheres^®^, NeutrAvidinTM labeled microspheres, 0.1 μm: excitation maximum 488 nm; emission maximum 605 nm; Invitrogen, ThermoFisher, Waltham, USA), resulting in a space between the two cover slips of less than 20 μm.

### Staining Antibodies

Primary monoclonal antibody 1H4 against human CD54 (ImmunoTools GmbH, Friesoythe, Germany), Alexa Fluor^®^ 594 Rabbit Anti-Mouse IgG (H+L) (Invitrogen, A-11062), mouse monoclonal antibody (mAb) anti-CD9 (Millipore, Billerica, MA), mouse mAb anti-human CD54 FITC-conjugated (ImmunoTools GmbH, Friesoythe, Germany), polyclonal antibody against human JAM-A [28] and secondary antibodies Alexa Fluor^®^ 568 and Alexa Fluor^®^ 647 were purchased from Invitrogen. 4Pi: Mouse monoclonal antibody (mAb) anti-CD9 (Millipore, Billerica, MA) or mouse mAb anti-human CD54 FITC-conjugated (ImmunoTools GmbH, Friesoythe, Germany) each stained with the secondary antibody Alexa Fluor^®^ 488 from Invitrogen.

### Fluorescence microscopy

Images were obtained with a commercial 4Pi microscope (TCS 4Pi microscope type A, Leica Microsystems, Wetzlar, Germany) employing oil immersion lenses (×100, numerical aperture 1.46). The TCS 4Pi is a confocal laser scanning microscope of type TCS SP2 incorporating single- as well as two-photon excitation, photon-counting by avalanche photodiodes, and a 4Pi attachment. Because of these features the microscope could be employed in the confocal mode with upright or inverted beam path and single- or two-photon excitation, or as a two-photon excitation 4Pi microscope. For those fluorophores which are not excitable in two-photon excitation, the microscope was used in confocal mode.

For confocal measurements single-photon excitation wavelengths of 488 and 561 nm were used, yielding best resolutions of 170 nm and 196 nm, respectively, in both x and y direction. The beam expander was set to 6. Fluorescence originating from the sample was passed through a filter cube (short-pass 700 nm, beam splitter 560 nm, band-pass 500–550 nm, and band-pass 607–683 nm) and its intensity was measured by photon-counting avalanche photodiodes (PerkinElmer, Massachusetts, USA).

For two-photon excitation, a mode-locked Ti:Sapphire Laser (MaiTai, Newport Spectra-Physics GmbH, Darmstadt, Germany) with pulse length stretched to 1.2 ps was used. The laser was tuned by a grating to a wavelength of 790 nm. The beam expander was set to 6. Detection tool and detection parameters are the same as in the confocal mode. Focus and phase of the counter-propagating beams were pre-aligned to the immobilized beads. Z-resolution was between 107 and 113 nm. Then xz-stacks of the cells were recorded with a pixel size between 13 x 13 nm and 15 x15 nm in xz-direction and a step size of 97 nm in y-direction.

Fluorescence microscopy for triple staining was performed on a confocal microscope LSM 780, equipped with a 63x objective, NA 1.4 (Carl Zeiss, Jena, Germany).

Raw images were linearly brightened, rescaled, and linearly filtered by a Gaussian Blur employing the image processing program ImageJ (Wayne Rasband, US National Institutes of Health, http://rsb.info.nih.gov/ij/). Ghost images, which arise in 4Pi microscopy because of side lobes of the point spread function (PSF), were eliminated by deconvolution using Leica software, which is based on a linear three-point or five-point deconvolution.

### CD9 Neutralizing Antibody experiment

For neutralizing antibody experiments purified mouse anti-human CD9 clone M-L13 (RUO)(BD Pharmingen^™^, 555370) or mouse IgG isotype control (Thermo Fisher, 02–6502) both in a final concentration of 20 [μg/ml] were added 1 hour before stimulation with TNF-α, which was added directly into the medium. The cells were fixed 24h later as described and analyzed by AFM.

### Statistical analysis

Results were considered significant when *p*-value < 0.05. Student‘s t-test or one-way ANOVA with a Bonferroni post-hoc analysis were performed where appropriate. Presented are the mean values ± SEM.

## Results

### Nanoscale protrusions on activated endothelia

**Fig 1:**
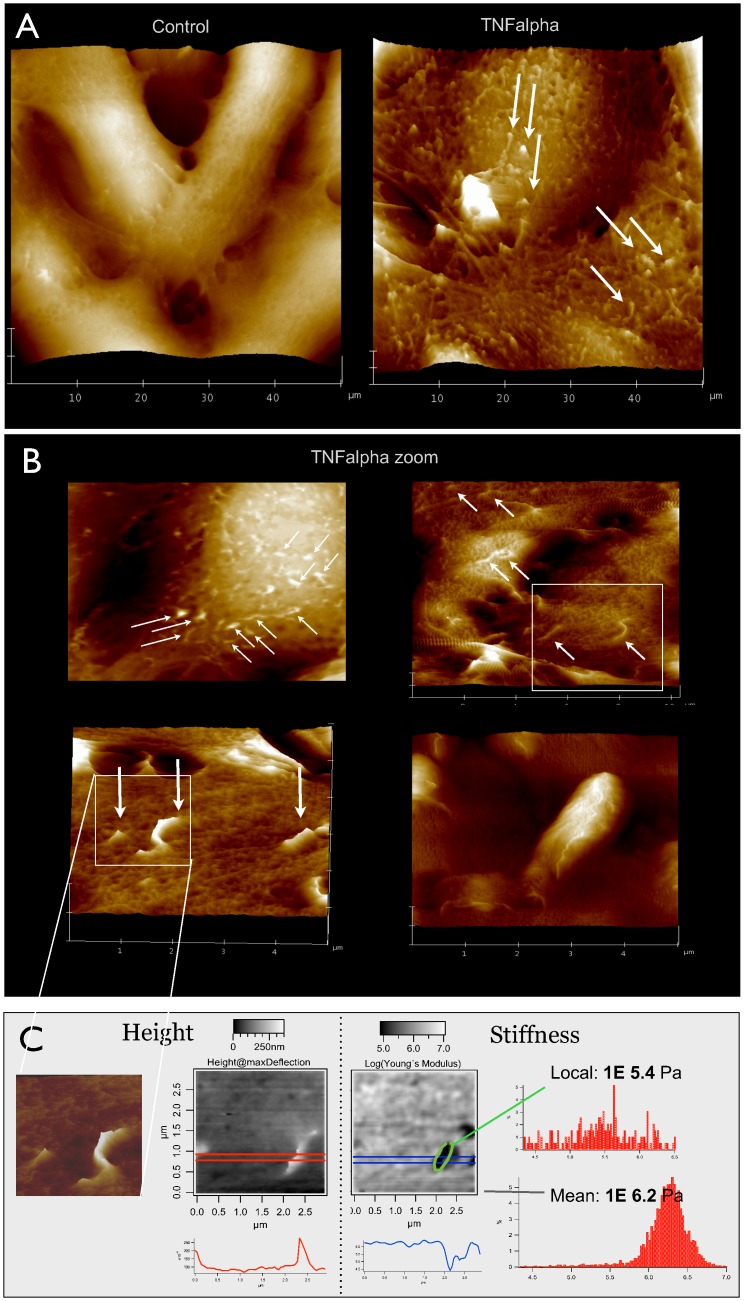
Nanoscale protrusions on activated endothelial cells. (**A**) AFM recordings (50 μm)^2^ on primary human endothelial cells (HUVEC) grown on permeable filter inserts show nice spindle-like morphology and a comparably smooth cell surface (*left*). Upon treatment with TNF-α [20 ng/ml] for 24 h, numerous protrusions appear and the surface becomes more rough (*right*). (**B**) Detailed morphology of protrusions after TNF-α stimulation is shown in higher resolution scans: (20 μm)^2^ (*upper left*) shows an almost uniform coverage with protrusions over the cell body, a (10 μm)² image (*upper right*) reveals single events and a (5 μm)^2^ zoom (*lower left*) shows a short straight and a long curved protrusion. Another 5 μm scan demonstrates a large item of 2 μm length and >300 nm diameter/height (*lower left*). (**C**) Mechanical stiffness analysis of the white marked window of B was performed on a (3 μm)^2^ area by recording force curves and re-plotting the calculated heights (*middle left*) or Young‘s moduli (*middle right*) in grey scale maps. Respectively, white color codes for either high or for soft areas. The height map is completely analogous to the contact mode image (*far left*), while the stiffness map shows dark areas at the site of the protrusion, which means mechanically soft. Accordingly, the profile plots below the maps demonstrate a protrusion height of 200 nm (*red line*) and a corresponding dip in mechanical stiffness to a minimum of 10E4.5 Pascal (*blue line*). The number values for Young‘s moduli are given as histograms (*far right*) for the region of interest (*upper diagram*) and for the total area under investigation (*lower diagram*). Pictures shown are representative for more than 10 independent cell preparations.

On TNF-α-activated, primary endothelia, we repeatedly observed membrane protrusions by AFM (Fig 1), especially, when the cells were grown on permeable filter membranes [21]. These protrusions typically measure 100–250 nm wide and up to 2 μm long. Distributed all over the cell, they showed a slight preference for lamellipodial regions. Among different cells there is considerable heterogeneity: Individual cells show a dense coverage with protrusions, while its neighbors often have a rather smooth surface. Protrusions can also be observed before activation, but only half as many.

Similar phenomena were observed in other primary isolates such as human microvascular (HMVEC) or mouse brain capillary endothelial cells (for mouse lymph node see [23]). However, cell lines like EAhy.926 (HUVEC-derived) or cerebEnd (mouse brain) failed to exhibit such distinct structures.

To investigate, how flexible the protrusive structures are, we performed mechanical stiffness mapping using the AFM (Fig 1C). An exemplary zoom on one protrusion demonstrates a significantly reduced Young‘s Modulus of 105.4 instead of 106.2 kPa. This is a factor of 6.3 softer than the surrounding mean cell stiffness. It can be concluded, that these protrusions are rather flexible structures, even though the geometric shape of a protrusion and the fact that the cells are slightly fixated can cause changes in stiffness [29–32].

### Microvilli contain ICAM-1

**Fig 2:**
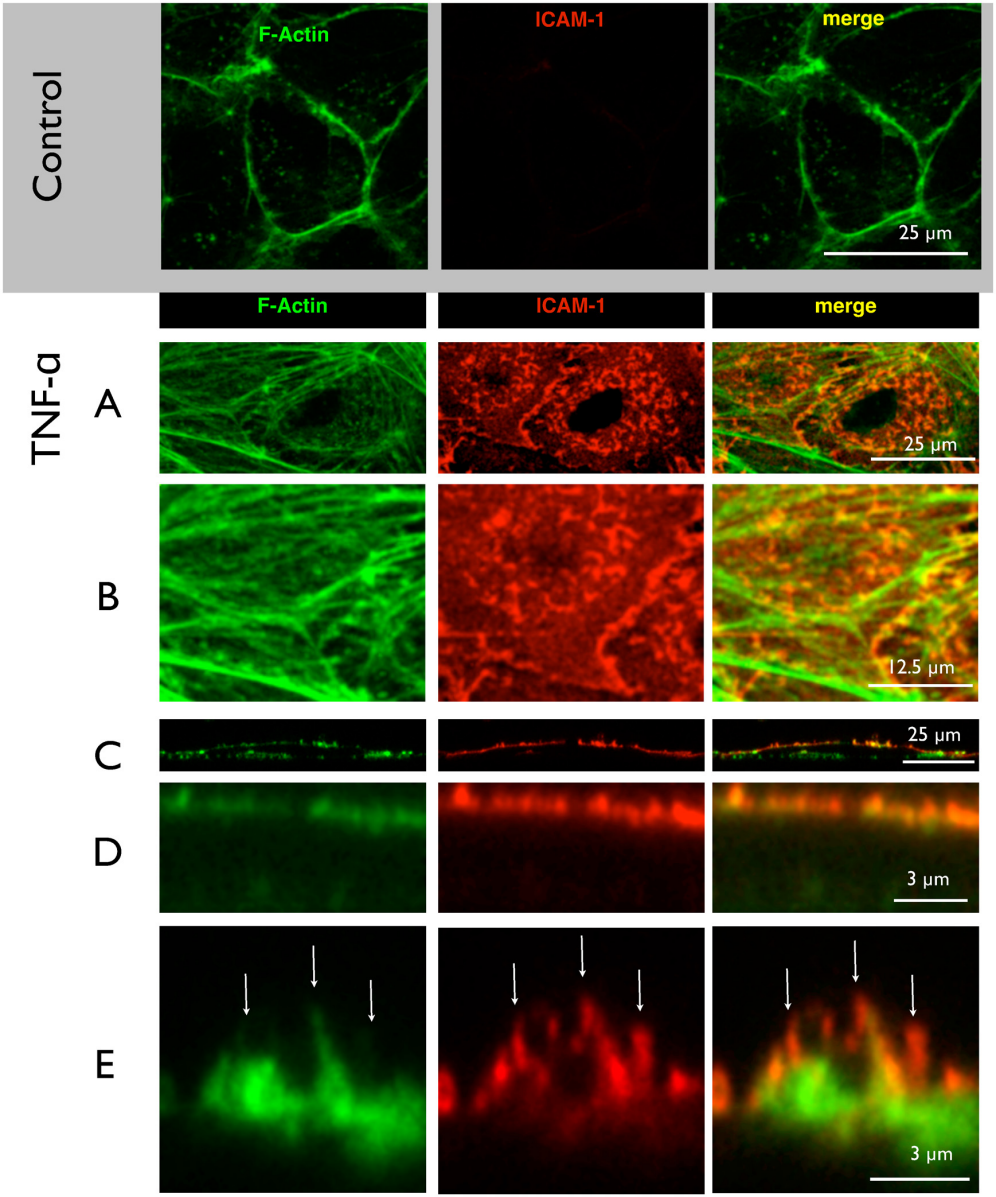
Immunostaining of ICAM-clusters. HUVECs before or after treatment with TNF-α were subjected to fluorescent staining for f-actin using phalloidin and immunolabeling for ICAM-1 using antibodies, respectively. The focal planes of confocal laser scanning microscopy (LSM) were adjusted to the apical surface. (**A**) represents a maximum intensity projection. ICAM-1 immunoreactivity is virtually absent from control cells and the actin forms a belt at the cell periphery along the junctions. Upon activation with (**TNF-α**), f-actin forms stress fibers (**A**), which exhibit many short debranchings as seen in the zoomed image (**B**). The ICAM-1 adhesion protein forms a punctuate pattern of elongated or even triangular spots with diameter of around 1 μm (**A**) They are distributed around the perinuclear area of the cell and are often located at the debranching sites (**B**). The reconstructed side view (**C**) shows f-actin both at the basal and apical areas, and ICAM-1 only at the apical surface. (**D**) A closer view on the apical surface demonstrates the clustered appearance and colocalization of ICAM-1 and f-actin. (**E**) A highest-resolution micrograph reveals a microvilli of more than 1 μm height which are decorated with ICAM-1 (white arrows). As seen from the merged image, ICAM-1 is mostly accompanied by f-actin but not the other way round. Images shown are representative for 3 independent cell preparations. In A and B, it is maximum intensity projections, while C-E are single slices in xz-plane (x = z scale).

In most studies, the term filopodia usually refers to horizontal extensions, i.e. substrate-exploring parts of the lamellipodium [33]. Structures spiking out of the apical membrane above the cell body have rarely been focused on. To classify whether they are rather pseudopodia (membrane blebs) or microvilli (f-actin stabilized) or cilia (microtubuli-containing), we performed (immuno)-cytochemistry for α-tubulin and f-actin (Fig 2). As expected, control cells (Fig 2E) did only show a very low basal immunoreactivity for ICAM-1, and a belt-like distribution of actin-strands at the cell borders. Upon activation with TNF-α, ICAM-1 spots arose in the perinuclear region. The nuclear region appeared dark, simply because it is vertically out of range when choosing a focal plane to represent the apical region. But the similarity between ICAM-1 pattern and the nano-architecture observed by AFM (Fig 1) was obvious. A-tubulin-staining did not show any structures above the cell surface, so that cilia were excluded (data not shown). But a f-actin staining demonstrated stress-fiber formation and a considerable number of short branches. The merged image revealed a high degree of colocalization of f-actin and ICAM-1. The spot-like appearance of adhesive clusters was supported by a side-view (Fig 2B), where it became clear, that stress fibers are located more basally and the adhesive spots strictly apically.

A zoom into the apical region revealed that not all ICAM-1 clusters were supported by cytoskeletal extensions. This observation argued for ICAM-1 clustering being prior to f-actin polymerization. Since the structures contain f-actin, but are negative for α-tubulin, they should be classified as microvilli.

### Cell Surface Nanoarchitecture

**Fig 3:**
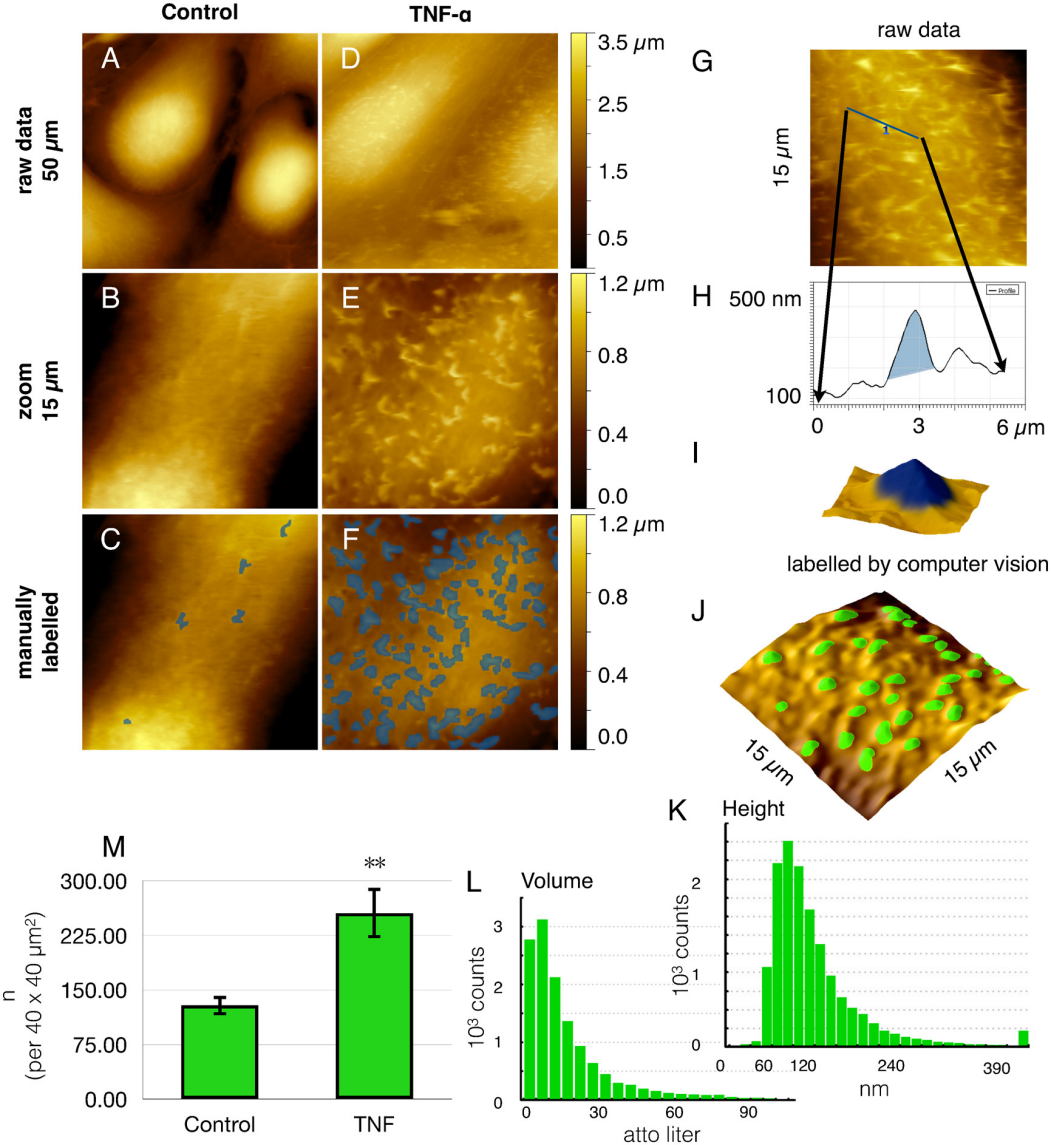
Quantitation of nanoscale surface protrusions. For morphological phenotyping of endothelial cell surfaces (HUVEC) were recorded by atomic force microscopy (AFM) without (**A**-**C**) or (**D**-**J**) after treatment with TNF-α. (**A**, **D**) An (50 μm)² overview demonstrates the cell bodies to become (**D**) longer and flatter after stimulation as compared to (**A**) controls, which exhibit a smoother surface. (**B**, **E**) Higher resolution to a (15 μm)^2^ scan reveals the microvilli, which have been manually marked blue in C and F, respectively. Here, contrasting examples are chosen for the sake of clarity. To achieve objectivity, computer vision was employed for the enumeration of nanostructures (**G**-**M**), referred to as nAnostic method. From raw data as in (**G**), profiles (**H**) and 3D-representations (**I**) were marked by an operator (*in blue*) to train the machine (supervised learning, for details see methods section). On the basis of identified objects, histograms of height (**K**) and objects‘ volume (**L**) were given for morphometrical profiling. Typical dimensions of the microvilli are 160 ± 80 nm height and a volume of 10–20 attoliter (*10E-18 l*). Finally, the increase of object count from 129 ± 14 objects / (40 μm)^2^ without to 260 ± 40 objects / (40 μm)^2^ with TNF-α clearly indicates cell activation(**M**). Shown are the mean values ± SEM of 20 images out of three independent experiments. ** p<0.*01 student’s t-test*.

To address the distribution characteristics under culture conditions identical to those for fluorescence imaging cells were seeded on glass cover slips—instead of permeable filter membranes—and subjected to AFM imaging (Fig 3). Overall, the HUVEC grew more elongated, but the phenomenon of microvilli formation was conserved also on non-permeable supports (Fig 3A and 3D), where the degree of apico-basal polarization is less (as compared to Fig 1). Consequently, the microvilli were fewer in number and they did not grow that long (Fig 3B and 3E). Nevertheless, they were clearly discernible as protruding objects (marked in blue by hand in Fig 3C and 3F)**.** The surface nano-architecture appeared completely analogous to the ICAM-1 pattern (as detected by immunofluorescence), given that the focal plane had been adjusted to the apical membrane (in Fig 2A and 2B).

### Quantitation of microvilli

With the need to fixate samples for AFM, it is impossible to conduct paired experiments. To be precise and as significant as possible in an unpaired setting, an operator-independent method for quantification was sought for. To obtain the count of nano-structures in an objective manner, we employed a computer vision procedure (Fig 3G–3M). Algorithms developed for AFM-topographies were implemented and trained to recognize the structures of interest (*machine learning)*. For AFM-topographies, this is a technological premiere. Automated pattern recognition did approximate the visual inspection very well: typically 85% of manually recognized objects. Biological heterogeneity was compensated for by increasing the experiment numbers: each condition was represented by 10 images (blindly addressed) at arbitrary regions of the sample in at least 3 independent runs (cell preparations). Each image was evaluated morphometrically (as indicated by histograms in Fig 3K and 3L) and the results were averaged (n > 30). Finally, the activation of endothelial cells by TNF-α led to a highly significant increase in the number of microvilli by 99% (Fig 3M).

### Tetraspanin CD9 coordinates the clustering of ICAM-1 and JAM-A

**Fig 4:**
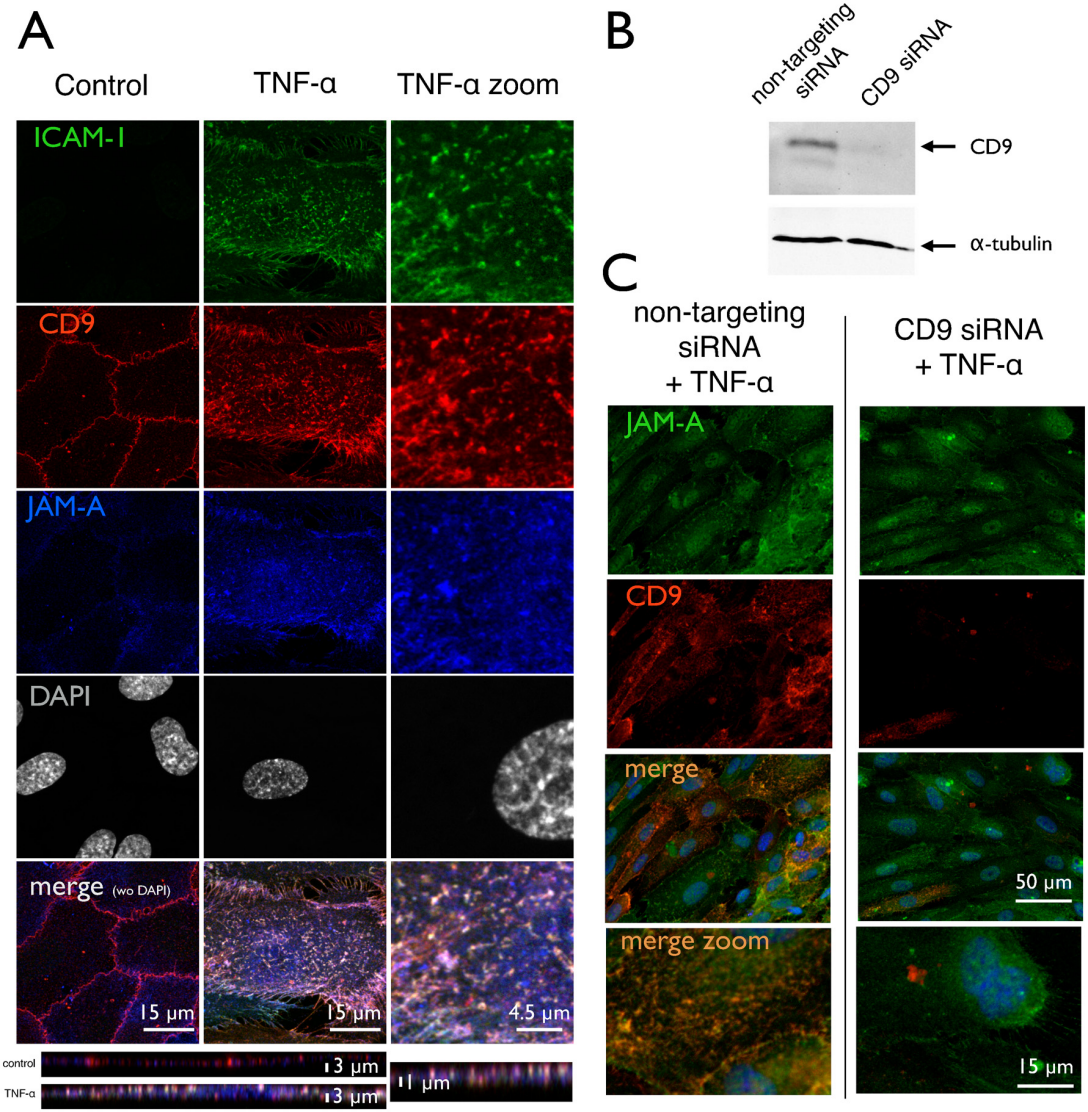
Association, clustering and redistribution of membrane proteins. HUVEC with or without treatment by TNF-α and CD9 siRNA were subjected to immunostaining for ICAM-1, tetraspanin CD9 and junctional adhesion molecule JAM-A, respectively. Images are taken by laser confocal microscopy (LSM) and projections in z are presented. (**A**) ICAM-1 is virtually absent from control cells, but is induced by TNF-α and forms a punctuate pattern of elongated or even kinked spots with a diameter of around 1 μm. They are distributed all over the cell surface with a preference for the perinuclear area. CD9, which is located at the cellular junctions in control cells becomes redistributed over the whole apical cell membrane upon cell activation—similar to the pattern of ICAM-1. JAM-A, which is often associated to CD9, exhibits the same behavior. In the activated cell state, there is a high degree of colocalization with both CD9 and JAM-A in the apical membrane clusters of ICAM-1—seen in the merged image as whitely spots. (**B**) Western blotting (WB) of CD9 in a siRNA-treated cell preparation as described in detail under methods section. Efficiency of siRNA-transfection was routinely controlled by WB for each umbilical vein preparation. (**C**) JAM-A immunoreactivity is hardly affected by CD9 siRNA-transfection; the expression level remains high, and the distribution still is mainly spread over the apical surface and not relocated to the cell junctions. CD9 protein level is vastly reduced as already confirmed in (**B**) by WB. The merged image demonstrates the reduced degree of clustering. Pictures shown are representative for more than 5 independent cell preparations.

The clustering of ICAM-1 is known to be coordinated by tetraspanins CD9 and CD81 [12]. Moreover, the junctional adhesion molecule-A (JAM-A), which has been implicated into leukocyte trafficking [34, 35], has also been assumed to integrate into EAPs [12]. This is confirmed here in immunofluorescence micrographs (Fig 4), where JAM-A was largely relocated from the cell border to the apical membrane compartment upon activation with TNF-α. Additionally, the distribution pattern changed from a continuous seam at the cell junction to a spotted appearance after activation. Moreover, JAM-A strongly co-localized with both CD9 and ICAM-1, as could be derived from the numerous white spots in the merged image. The elevation of CD9 and ICAM-1 clusters was also confirmed by 4Pi microscopy (S4 Fig). The redistribution of JAM-A has been reported without looking at ICAM-1 and CD-9 [36], albeit JAM-A was considered to open cell-cell-junctions during endothelial activation [22, 37].

### Tetraspanin CD9-dependent clustering drives microvilli formation

**Fig 5:**
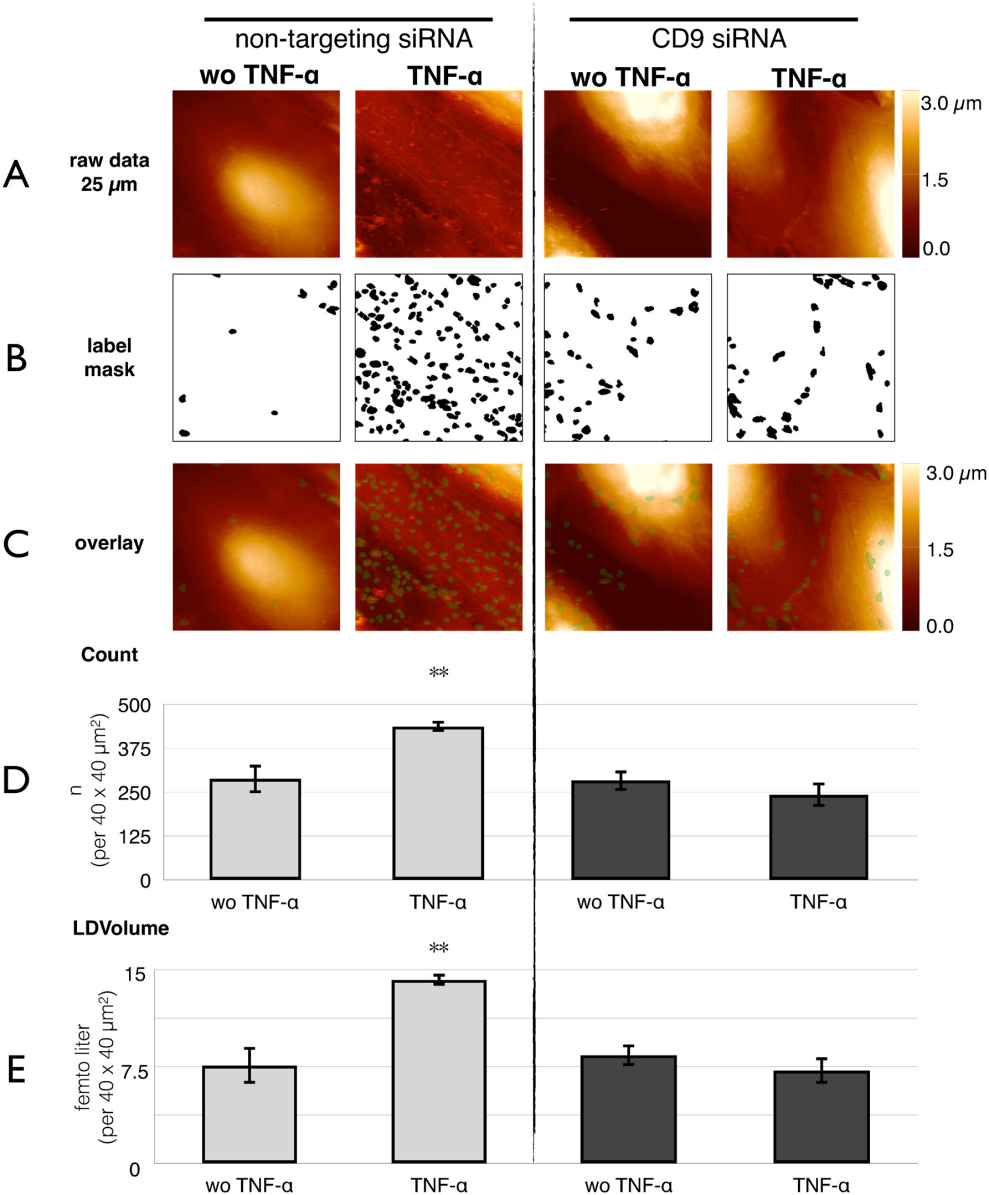
Inhibition by CD9-siRNA of microvilli formation. HUVECs treated with CD9 siRNA or non-targeting siRNA either stimulated with TNF-α or without TNF-α, respectively, were subjected to quantitative AFM-analysis.(**A**) Surface topography is recorded at 10 arbitrary areas of the sample (one representative is shown here). (**B**) Raw data were subjected to computer vision as demonstrated in Fig 3 to identify microvilli. The result is presented as a black and white mask. (**C**) For better visualization, an overlay of A and B is given. (**D**) The object count rises from 290 ± 40 objects / (40 μm)^2^ without to 437 ± 16 objects / (40 μm)^2^ with TNF-α at non-targeting siRNA. This morphometric change is abrogated by pretreatment of the cells with CD9 siRNA. Here, the object count without TNF-α 280 ± 30 objects / (40 μm)^2^ or with TNF-α 240 ± 40 objects / (40 μm)^2^ is not significantly different. (**E**) Similar is for the size, measured as „local differential volumes”(LDV) of the microvilli: The increase from 7.6 ± 1.4 *f* l / (40 μm)^2^ to 14.2 ± 0.5 *f* l / (40 μm)^2^ (by 87% after TNF-α) is completely abolished in CD9 knock-down cells; without TNF-α 8.4 ± 0.9 *f* l / (40 μm)^2^ is not significantly different to TNF-α 7.2 ± 1.1 *f* l / (40 μm)^2^. Shown are the mean values ± SEM of 30 images out of three independent experiments. **p<0.*01 post-hoc Bonferroni analysis compared to each bar*.

To test whether clustering of CD9 and ICAM-1 is upstream of microvilli formation, the clustering was inhibited by siRNA directed against CD9 expression (Fig 5). Already in non-stimulated cells, which were transfected with non-targeting siRNA as control (Fig 5D, *far left panel*), the number of microvilli was higher than under naïve (without siRNA) conditions (Fig 3M). This inevitable pre-stimulation of the cells might be due to the siRNA incubation protocol. Nevertheless, the increase after stimulation with TNF-α holds true (middle left panel), even though the effect was smaller (only 50% increase). When pre-incubated with CD9-siRNA, the number of microvilli did not change as compared to the mock-transfected controls (middle *right panel*). So, in resting cells, CD9-knock down had no effect. However, when TNF-α was added, no increase was provoked anymore (*right panel*). The values for *local deviational volumes* (LDV) are analogous to the object count. Supplementary, we could prove the inhibition of microvilli formation by the CD9 antibody ML-13 (S3 Fig). The CD9 antibody inhibited the effect of TNF-α by 60%.

The abrogation of effect by both the CD9 siRNA and the CD9 antibody ML-13 clearly shows a leading role of CD9 in the formation of microvilli.

Also, a siRNA against JAM-A was generated and proven effective [28]. However, the JAM-A siRNA failed to reduce the number of microvilli formation as detected by AFM (data not shown). Obviously, JAM-A is rather passively associated to EAPs.

### Modeling nanoscale precursors of the docking structure

**Fig 6:**
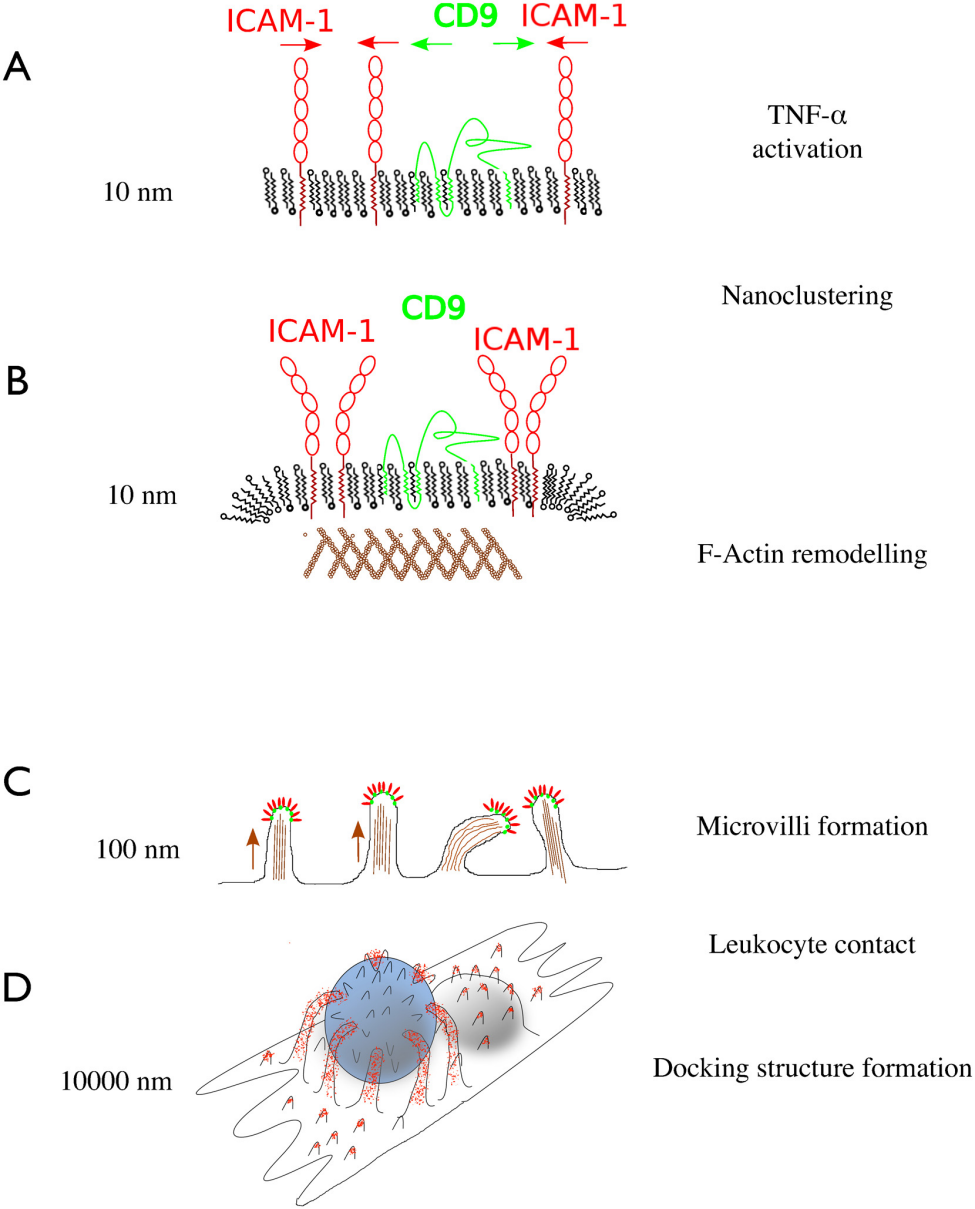
Schematic model of adhesive microvilli formation. (**A**) Upon activation of endothelial cells, ICAM-1 is upregulated and processed into the plasma membrane; it associates with tetraspanin CD9 to form clusters 2-dimensional. (**B**) The clusters (or: endothelial adhesive platforms EAPs also contain JAM-A, which is not depicted here) recruit f-actin, potentially through a RhoG dependent mechanism. (**C**) The clustered adhesion platforms are propelled upwards by typically 160 ± 80 nm, thereby increasing the interaction probability with leukocytes. (**D**) Upon leukocyte contact, the microvilli are further elongated to develop a full docking structure with long filopodia engulfing the leukocyte.

Taking the results together, a model emerges, how ICAM-1 enriched microvilli are formed in a sequence of steps (Fig 6): Upon stimulation with TNF-α, ICAM-1 is upregulated and translocated into the apical plasma membrane (Fig 6A). ICAM-1 associates with tetraspanin CD9-enriched domains to form functional clusters (EAPs). JAM-A is associated as well, but omitted here for the sake of simplicity. These ICAM-1 clusters (EAPs) recruit f-actin nucleation or branchings by a Rho-dependent mechanism discussed below (Fig 6B). The f-actin spots are polymerized to protrude 160 ± 80 nm (SD) above mean surface level—prior to any leukocyte contact (Fig 6C). An ICAM-1 decorated protrusion is suited for grasping leukocytes, because it is protruding, flexible and adhesive (in other words: 160 ± 80 nm high (Fig 3), relatively soft (Fig 1) and decorated with ICAM-1 (Fig 2). Microvilli being engaged in leukocyte binding, are putatively elongated to maturate into a docking structure (Fig 6D).

## Discussion

The present study shows quantitatively, that endothelial cells do not only induce expression of adhesion molecules like ICAM-1 upon stimulation with TNF-α, but also adapt their surface architecture through the formation of microvilli. These microvilli are preformed by endothelial cells before any leukocyte contact [1, 9, 16, 38].

A cytoskeletal response to TNF-α in the absence of leukocytes has been described earlier in a study on small GTPases Rho, Rac, cdc42 [39]. Using fluorescence-microscopy, the authors reported on the formation of f-actin spots or short branchings out of the lamellipodium (in a horizontal way), which are called microspikes [40, 41] and they could show a colocalization of moesin, ezrin and ICAM-1 [25, 26]. This process involves the cytoskeletal regulators Rho and Rac, but not cdc42 [42]. The force-generating myosin and its kinase MLCK (myosin light chain kinase) are closely involved in leukocyte transmigration, too [22, 43]. They have at least two functions: (i) relaxing the endothelial cytoskeleton and (ii) pulling down engulfed leukocytes trough the contraction of docked membrane arms [44, 45].

Even the formation of longer protrusions on the apical membrane has been observed after activation with TNF-α. Interestingly, in a non-endothelial cell model of Cos7 cells, ICAM-1 over-expression induced the generation of filopodia-like structures observed qualitatively using scanning electron microscopy [15]. This is one of the rare cases, when investigators refer to apically located protrusions—in contrast to the substrate-exploring extensions at the leading edge using a height sensitive method.

A study by van Buul elegantly answered the question, whether ICAM-1 is attracted to existing „cytoskeletal spikes”or if ICAM-1 clustering induces polymerization of f-actin at this particular spot. The authors found the filopodia formation markedly reduced when a truncated version was transfected instead of the wild-type ICAM-1 [15]. This observation clearly demonstrates that the actin remodeling is driven by ICAM-1.

Various downstream effectors of ICAM-1 and Rho have been identified. Among the signaling molecules involved in the development of docking structures are RhoA, ROCK, MLCK, filamin, cortactin, and ERM (ezrin, radicin, moesin), to name the most important [46, 47]. A parallel pathway hints at src-kinase and pyk-2, as candidates for the generation of protrusions [48]. How ICAM-1 clusters exactly orchestrate their self-elevation will be subject to future studies.

Little is known about processes upstream of ICAM-1 clustering, because for most experimental models, the binding of leukocytes (or LFA1-coated microspheres) axiomatically sets the starting point. For example, Barreiro et al found ICAM-1 to be recruited to pre-existing tetraspanin-enriched membrane domains, which function as endothelial adhesive platforms (EAPs), upon binding of lymphocytes [12]. Surprisingly, recruitment of ICAM-1 into EAPs was independent of LFA-1 binding as well as independent of actin-anchorage. Moreover, CD9 had a leading role in the clustering (EAP-formation) and it was more related to ICAM-1 than VCAM-1. Since in FRAP experiments the cytoplasmic tail-truncated ICAM-1 had a faster recovery rate at the plasma membrane than wild-type ICAM-1 and since docking structures are only built by actin-anchored endothelial adhesion molecules it can be concluded that ICAM-1 interacts with the cytoskeletal f-actin.

Furthermore, transfection of unstimulated HUVECs with exogenous ICAM-1 (pCDM8-ICAM-1) and incubation with anti-ICAM-1-antibodies was sufficient for the induction of stress fibers. It means that clustering at the membrane is sufficient for cytoskeletal changes, i.e. actin stress fibers [25]. These observations are in good accordance with our results, because CD9 siRNA abrogates microvilli formation here. Consequently our results indicate that clustering of ICAM-1 in a CD9 dependent manner is not only sufficient but also needed for the formation of microvilli.

The mechanism of how CD9 contributes to microvilli formation has been worked on in recent years. In oocytes Runge et al. demonstrated the relevance of CD9 for the ultrastructural integrity of microvilli by TEM and analyzed the co-immunoprecipitation of CD9 with the two Ig superfamily cis partner, EW-2 and EWI-F [49]. It was shown in 2011, that the C-terminal tail of CD9 is highly relevant for the formation of microvilli and cell adhesion [50].

In this study, inhibition of microvilli formation by the CD9 antibody ML-13 was proven to be effective (S3 Fig). The extracellular domain of CD9 is thought to be responsible for clustering as well for homodimerization as for the interaction with ICAM-1 [12, 51, 52]. The antibody might sterically block these interaction sites.

The biological relevance of this nano-feature has been nicely demonstrated in a study, where CD9 antibody ML-13 inhibited the transmigration of monocytes through brain endothelial cells [53]. A simple explanation for this phenomenon is given by reduced accessibility of the attachment sites as outlined in the following geometrical model.

**Fig 7:**
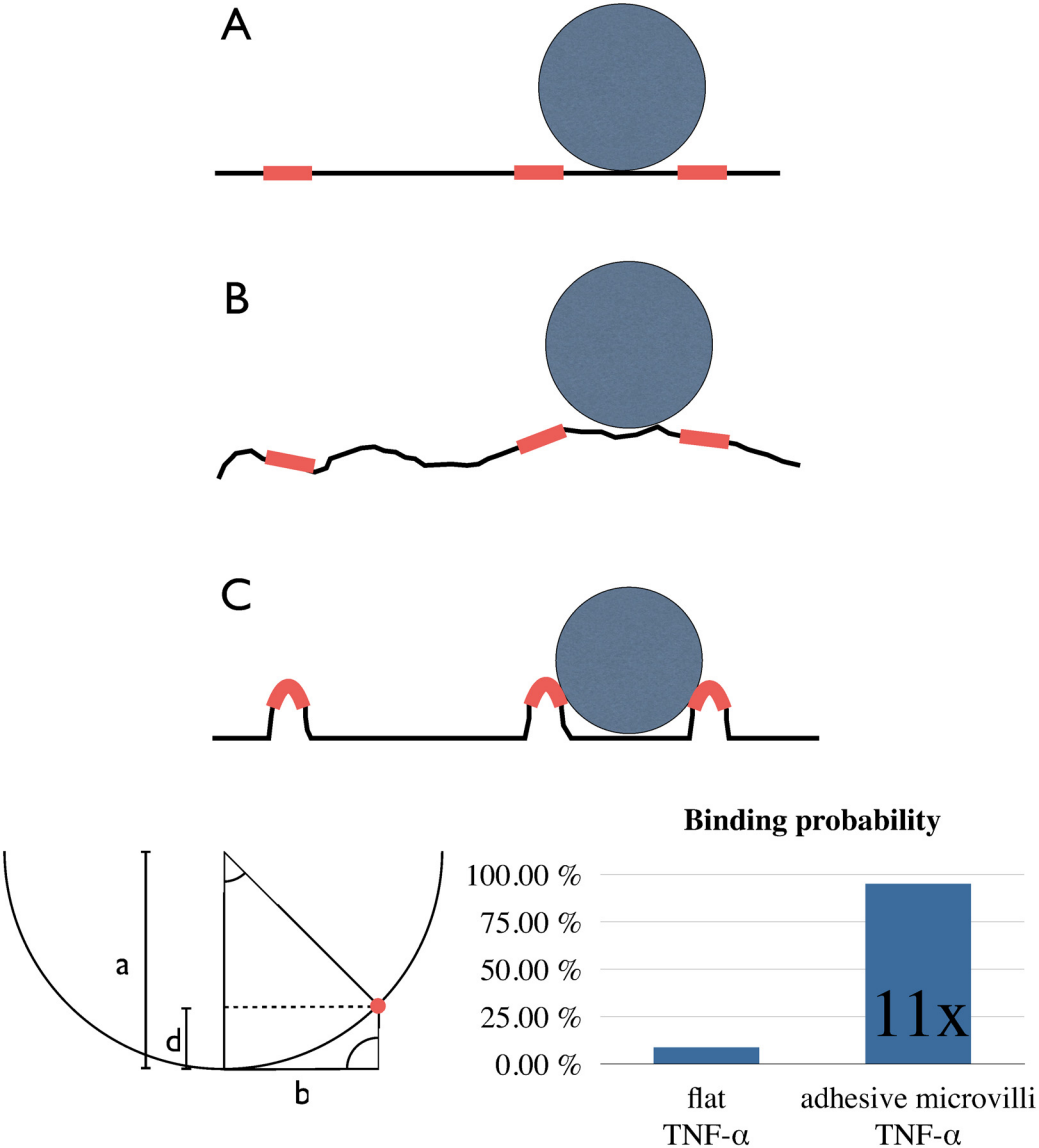
Sketch on physiological relevance of adhesive microvilli. Geometrical considerations illustrate the impact of microvilli on binding probability of leukocytes. General assumptions are: ICAM-1 clusters (*adhesive platforms*, *EAP*) cover 10% of the endothelial surface and are equally distributed at mean distance of 2b = 2.5 μm, and leukocytes have smooth, spherical shape, radius a = 5 μm. (**A**) In a simple model of a flat endothelial surface, the chance of a leukocyte to meet an EAP upon first contact just equals the ICAM-1 surface coverage (10%). (**B**) With an arbitrary convolvement of membrane surface as in real cells (cytoskeletal roughness), many EAPs become inaccessible for leukocytes. The binding probability decreases close to zero, depending on the degree of surface roughness. (**C**) This sterical shielding effect is avoided, when EAPs are elevated above ground level. With the geometrical assumptions made above, EAPs reach a binding probability of 95% when lifted upwards by 160 nm.

To estimate the functional relevance of microvilli formation, we would like to add a geometrical aspect (Fig 7). The regions of the vasculature, where diapedesis takes place, are very thin (capillaries or venules (10–50 μm)) and leukocytes are frequently in touch with the endothelium. For the sake of simplicity, the following assumptions are made for calculating the binding efficiency: the surface coverage with ICAM-1 is 10% of the area (clusters are equally distributed). The leukocyte is circular and has a smooth surface. Modeling three geometrical cases helps to understand how the corrugation of the endothelial surface affects the binding probability of leukocytes.

On a flat surface (Fig 7A), the binding probability is equal to the ligand coverage (here: 10%).

When the surface is convolved randomly (Fig 7B), then most of the adhesive ICAM-1 spots are „hidden”in the valleys, so that the access for the leukocyte is sterically hindered. Only few clusters coincide with hilltops of the surface. As a result, the binding probability is reduced through surface convolvement.

When ICAM-1 clusters are „intentionally”propelled upwards (Fig 7C), the likelihood of contact is vastly enhanced (by factor 11 in our model calculation). Assuming straight microvilli and circular leukocytes, the height threshold necessary for 100% binding efficiency is 167 nm. This is in perfect accordance with the measured value of 160 ± 80 nm. Of course, the leukocyte has many protrusions and LFA1 is not distributed equally. But still, with all the limitations of this simplified model, the main issue will remain valid: the formation of microvilli probably enhances capture efficiency.

The importance of the cytoskeleton for leukocyte binding has already been proven experimentally in a former study by Wojciak-Stothard and coworkers [39]. Inhibition of Rho did not alter the expression level of adhesion molecules PECAM, ICAM-1 and VCAM, but did reduce the adhesion of monocytes. The authors concluded: “The assembly of cytoskeletal connections with the clusters of membrane receptors could then provide “footholds”for attached monocytes and create the tension required for their spreading and migration”[39]. Indeed, this did exactly predict our results [24], when TNF-α increased not only the diapedesis, but also the adhesion and lateral speed of non-stimulated/quiescent leukocytes on the endothelium.

It is tempting to speculate that not only the surface architecture but also a complete transmigratory channel might be preformed: TMCups are in a dynamic balance with a vesicle pool and close again within a time course of one minute [54], This vesicle pool is associated with vesiculo-vacuolar organelles [55], and/or a lateral border compartment [22]. All these phenomena favor preformed channels to exist. Indeed, a recent, highly interesting study by Lemichez et al. prove not only a local softening at putative diapedesis sites [23], but even the formation of complete transcellular channels in HUVEC upon administration of bacterial toxin [56]. No leukocytes were used in the study. These observations not only imply an actively leading role of the endothelium during leukocyte diapedesis but also for inflammatory side effects like edema formation. However, we did not observe any formation of channels upon administration of TNF-α.

## Conclusion

In conclusion, nanoscale membrane architecture offers a new functional aspect: elevating the ICAM-1 clusters into direction of the bloodstream facilitates leukocyte capture. The advantage is twofold: an energy-consuming protein expression (ICAM-1) is minimized and the immune system responds faster. This mechanism may have evolved to render the immune response more efficient. That the microvilli formation is conserved *in vitro*, hints at its elementary importance for vascular biology. The future may see nanostructure quantitation emerging as a sensitive tool for studying inflammatory activation on a cellular basis.

## Supporting Information

S1 Fig Preformed circular, ICAM-1 positive structure. HUVECs were stimulated with TNF-α and (**A**) stained for ICAM-1 (*red*) and f-actin (*green*). Several slices of confocal microscopy were used to reconstruct a 3D-View. Hereby, contrast enhancement and gaussian blurring were applied. It shows a circular ICAM-1 positive structure of 1 μm, which is partially stabilized with f-actin. (**B**) Similar arrangements of membrane protrusions are also found by atomic force microscopy.
(TIF)

S2 Fig Effect of CD9 siRNA on ICAM-1 clustering. HUVECs were treated either with non-targeting siRNA or with CD9 siRNA and then stimulated with TNF-α. (**A**) Fluorescence Images of ICAM-1 were analyzed by image processing. (**B**) Briefly, cluster analysis was performed using the software Ilastik 0.5. The black spots representing ICAM-1 clusters are given in a binary map. (**C**) Pretreatment with CD9 siRNA reduces the number of ICAM-1 clusters from 8700 ± 800 objects / (225 μm)^2^ to 5900 ± 500 objects / (225 μm)^2^ (by 32%). The images are representative for 27 images in two independent runs and presented are the mean values ± SEM.
(TIF)

S3 Fig Preincubation with CD9 antibody. HUVECs pretreated with the CD9-antibody (ML-13) or unspecific IgG were stimulated with TNF-ɑ and subjected to AFM-nanoanalysis. Automated quantification (**A**) revealed an increase of microvilli from 88 ± 6 objects / (40 μm)^2^ to 260 ± 20 objects / (40 μm)^2^ by TNF-ɑ in the unspecific IgG control (n = 3). CD9-antibody ML-13 (without TNF-ɑ 77 ± 6 objects / (40 μm)^2^) leads to reduced microvilli formation of 151 ± 6 objects / (40 μm)^2^ (inhibition by 60%). Representative atomic force microscope images (**B**) of either unspecific IgG or CD-9 antibody (ML-13) visualizing the automatically analyzed data of (**A**). * p<0.05 post-hoc Bonferroni analysis.
(TIF)

S4 Fig 4Pi-microscopy of ICAM-1 and CD9. HUVECs pretreated with TNF-ɑ were either stained for ICAM-1 (**A**) or CD9 (**B**) and 4Pi microscopy, reaching an axial resolution of 107–113 nm. Thereby it could be shown that ICAM-1 and CD9 clustering leads to an elevation of the clusters above the cell membrane level. (**C**) Deconvolution algorithms were used to process the 4Pi images. The raw data of the 4Pi microscope contain so-called ghost images due to the induced interference of the exciting laser. Using fluorescent beads of subresolution size (TransFluorSpheres 0.1 μm, Molecular Probes) the point spread function (PSF) can be approximated and the images might be deconvolved. This reduces the intensity of the side lobes (green triangle). Due to phase shift and sample geometries the deconvolved image may still contain interference phenomena.
(TIF)
