# Supplementary figures and images for: Nanoscale Imaging Reveals a Tetraspanin-CD9 Coordinated Elevation of Endothelial ICAM-1 Clusters
Source: PLoS One. 2016 Jan 5;11(1):e0146598. doi: 10.1371/journal.pone.0146598 (PMC4701507; doi:10.1371/journal.pone.0146598)

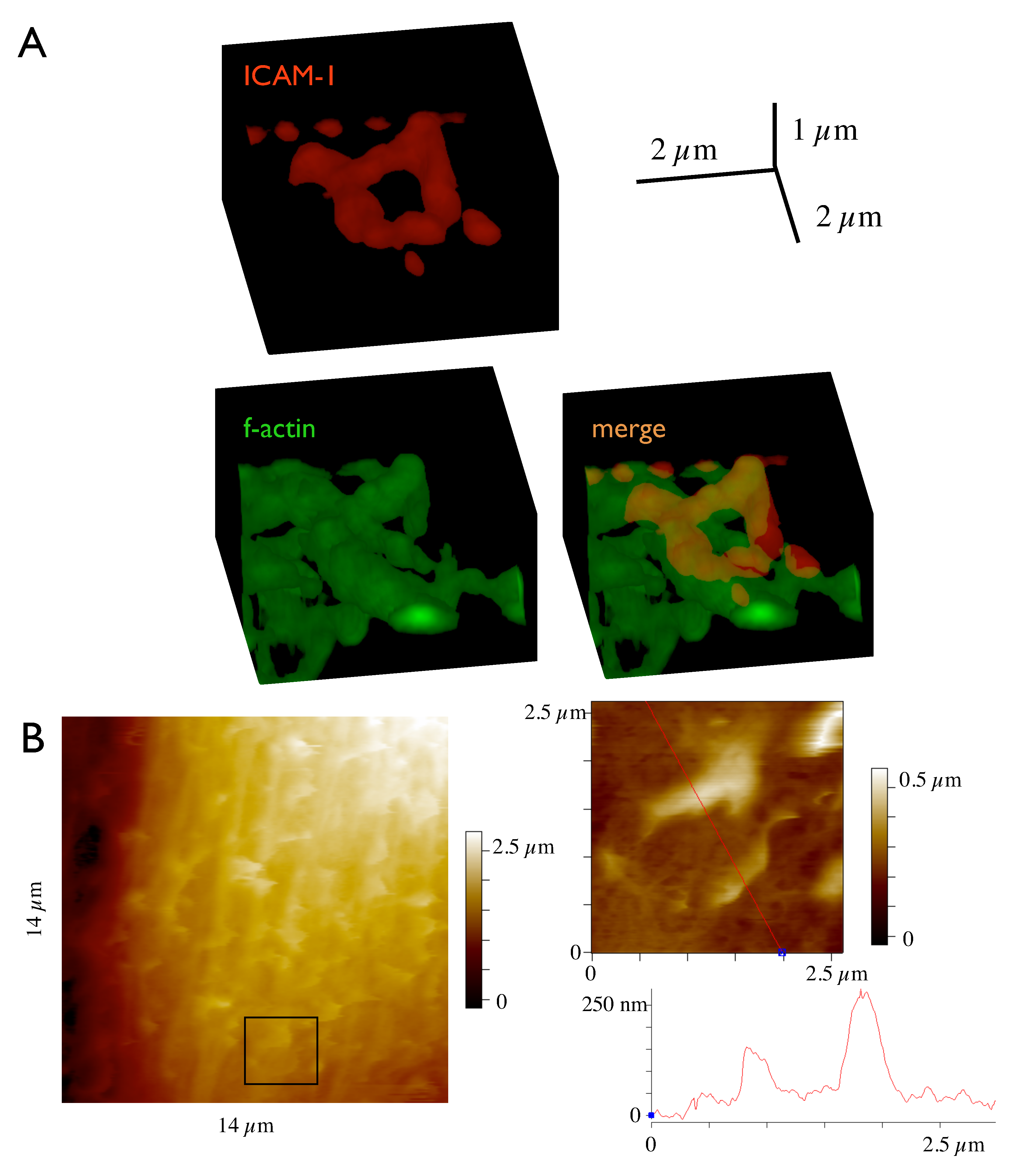

Supplement: S1 Fig — HUVECs were stimulated with TNF-α and (A) stained for ICAM-1 (red) and f-actin (green). Several slices of confocal microscopy were used to reconstruct a 3D-View. Hereby, contrast enhancement and gaussian blurring were applied. It shows a circular ICAM-1 positive structure of 1 μm, which is partially stabilized with f-actin. (B) Similar arrangements of membrane protrusions are also found by atomic force microscopy. (TIF) [file pone.0146598.s001.tif]

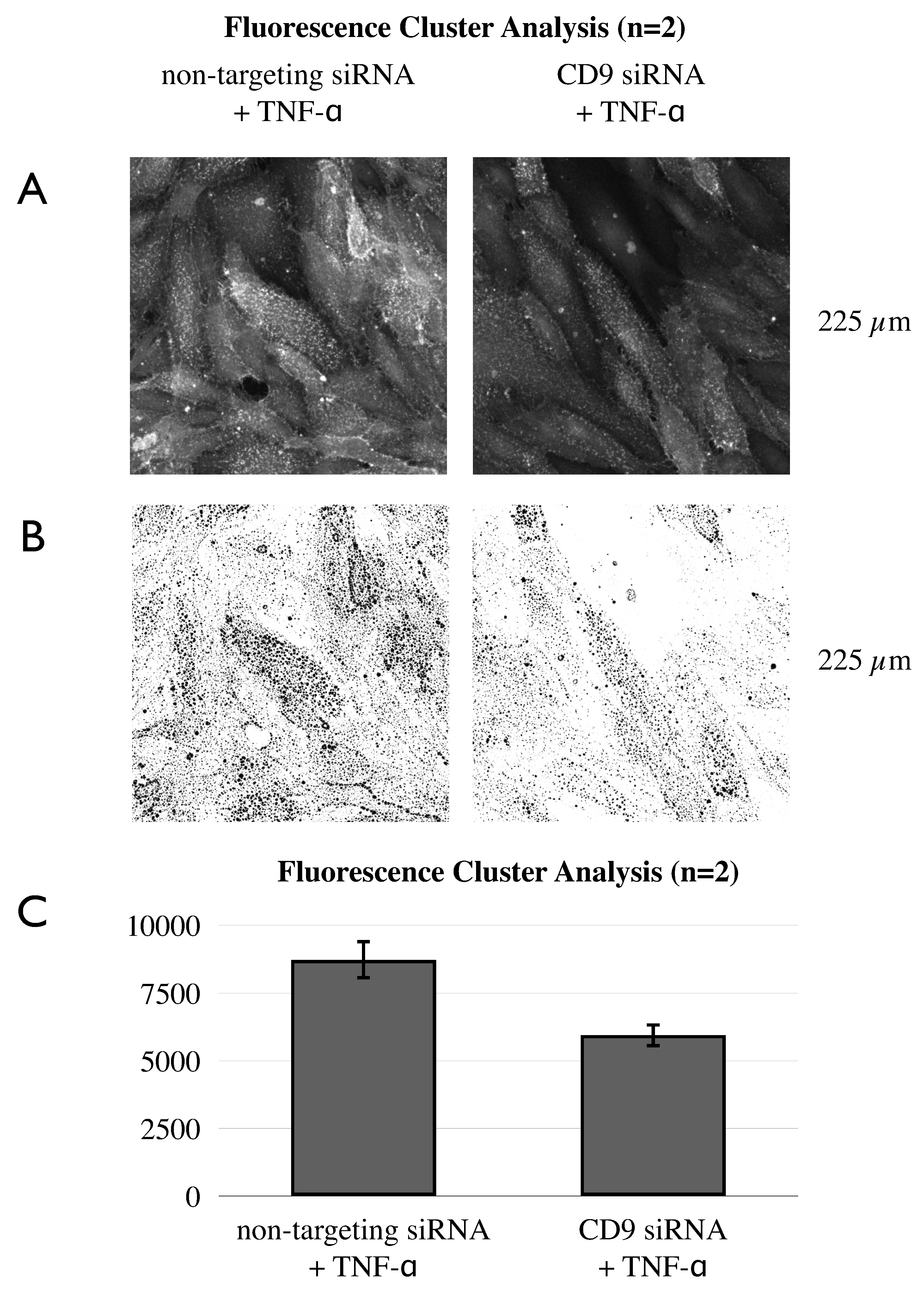

Supplement: S2 Fig — HUVECs were treated either with non-targeting siRNA or with CD9 siRNA and then stimulated with TNF-α. (A) Fluorescence Images of ICAM-1 were analyzed by image processing. (B) Briefly, cluster analysis was performed using the software Ilastik 0.5. The black spots representing ICAM-1 clusters are given in a binary map. (C) Pretreatment with CD9 siRNA reduces the number of ICAM-1 clusters from 8700 ± 800 objects / (225 μm)2 to 5900 ± 500 objects / (225 μm)2 (by 32%). The images are representative for 27 images in two independent runs and presented are the mean values ± SEM. (TIF) [file pone.0146598.s002.tif]

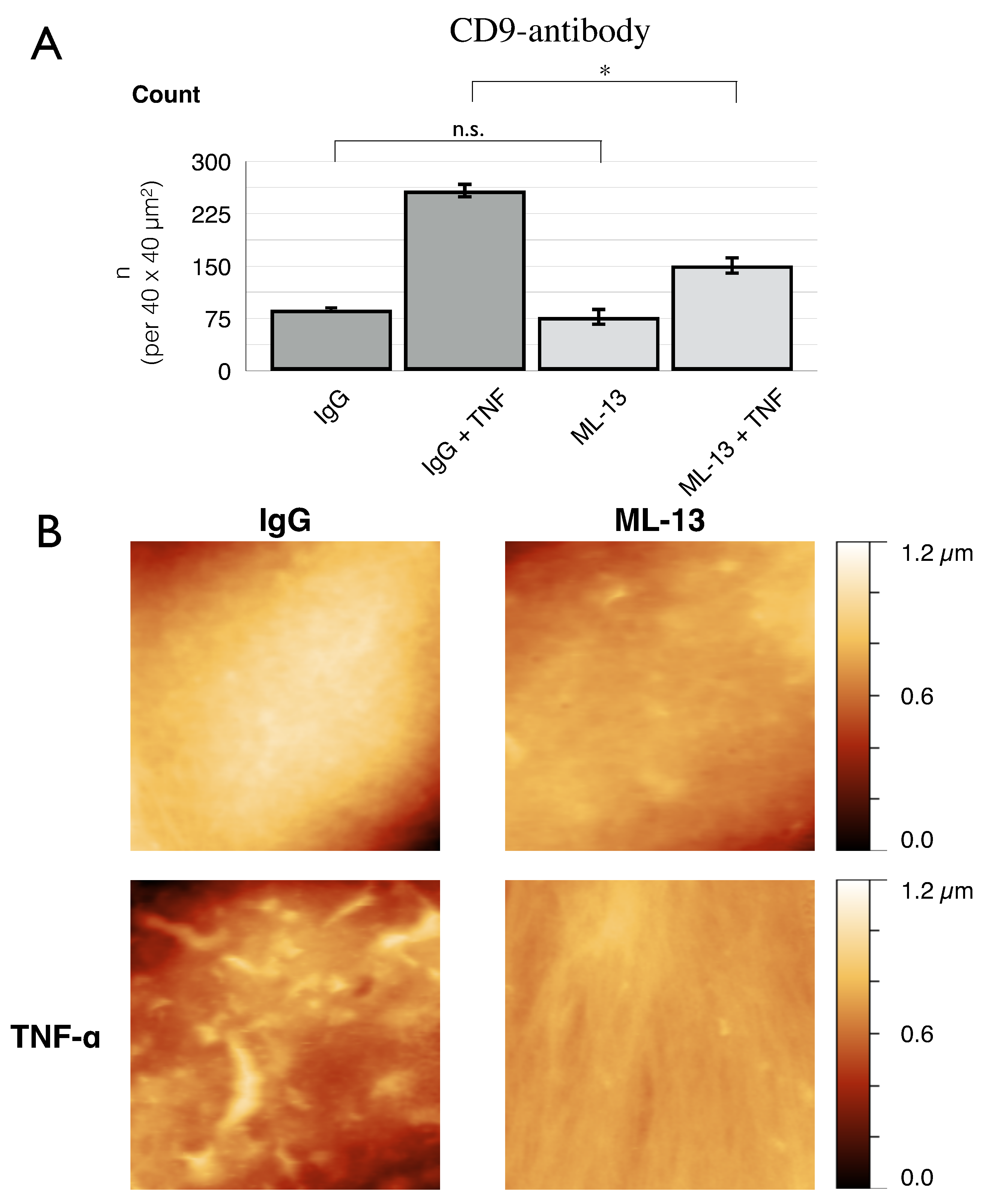

Supplement: S3 Fig — HUVECs pretreated with the CD9-antibody (ML-13) or unspecific IgG were stimulated with TNF-ɑ and subjected to AFM-nanoanalysis. Automated quantification (A) revealed an increase of microvilli from 88 ± 6 objects / (40 μm)2 to 260 ± 20 objects / (40 μm)2 by TNF-ɑ in the unspecific IgG control (n = 3). CD9-antibody ML-13 (without TNF-ɑ 77 ± 6 objects / (40 μm)2) leads to reduced microvilli formation of 151 ± 6 objects / (40 μm)2 (inhibition by 60%). Representative atomic force microscope images (B) of either unspecific IgG or CD-9 antibody (ML-13) visualizing the automatically analyzed data of (A). * p<0.05 post-hoc Bonferroni analysis. (TIF) [file pone.0146598.s003.tif]

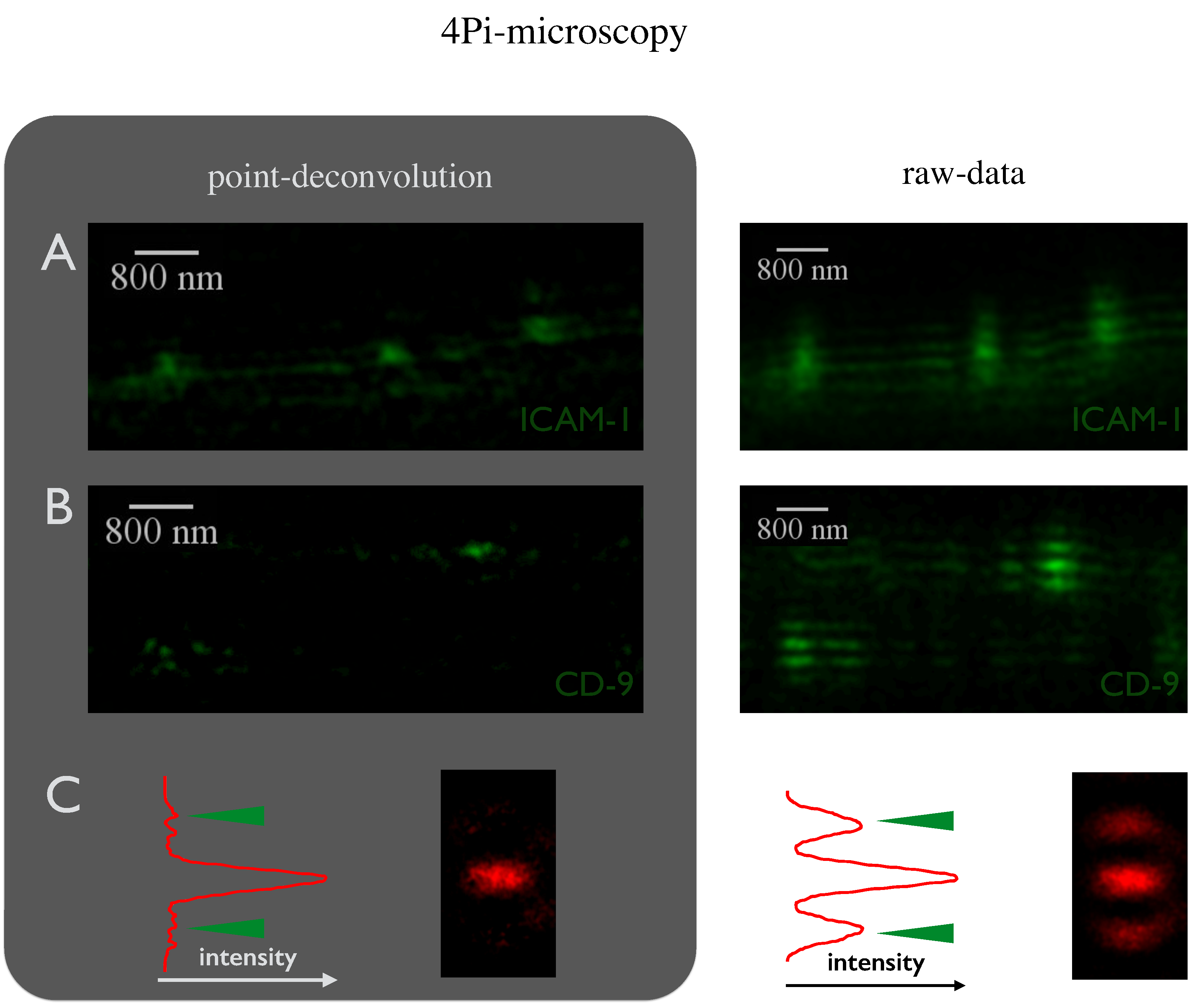

Supplement: S4 Fig — HUVECs pretreated with TNF-ɑ were either stained for ICAM-1 (A) or CD9 (B) and 4Pi microscopy, reaching an axial resolution of 107–113 nm. Thereby it could be shown that ICAM-1 and CD9 clustering leads to an elevation of the clusters above the cell membrane level. (C) Deconvolution algorithms were used to process the 4Pi images. The raw data of the 4Pi microscope contain so-called ghost images due to the induced interference of the exciting laser. Using fluorescent beads of subresolution size (TransFluorSpheres 0.1 μm, Molecular Probes) the point spread function (PSF) can be approximated and the images might be deconvolved. This reduces the intensity of the side lobes (green triangle). Due to phase shift and sample geometries the deconvolved image may still contain interference phenomena. (TIF) [file pone.0146598.s004.tif]
